# Education Research: Career, Research, and Mentorship Outcomes of an International Neuroimmunology Education Exchange Program

**DOI:** 10.1212/NE9.0000000000200374

**Published:** 2026-10-09

**Authors:** Cassia Elisa Marin, Bruna Leles Vieira de Souza, Vanessa Fernanda Moreira Ferreira, Joao Vitor Mahler, Nathane Braga, Milena Sales Pitombeira, Natalia Talim, Maria Fernanda Mendes, Vanessa Daccach Marques, Alfredo Damasceno, Antonio Pereira Gomes Neto, Maria Lucia Brito Ferreira, José Artur Costa D Almeida, Silvia N. Tenembaum, Xavier Montalban, Daniel Ontaneda, Dagoberto Callegaro, Jefferson Becker, Marco A. Lana-Peixoto, Marcelo Matiello

**Affiliations:** 1 Universidade de Caxias do Sul, Caxias do Sul, Brazil;; 2 Department of Neurology, Mass General Brigham, Harvard Medical School, Boston, MA;; 3 Hospital Universitário Antonio Pedro, Universidade Federal Fluminense (HUAP-UFF), Niterói, Brazil;; 4 Hospital Geral de Fortaleza (CIAPEM-HGF), Brazil;; 5 CIEM MS Research Center, Faculdade de Medicina da Universidade Federal de Minas Gerais, Belo Horizonte, Brazil;; 6 Santa Casa de São Paulo, Brazil;; 7 Hospital das Clínicas da Faculdade de Medicina de Ribeirão Preto - Universidade de São Paulo (HCFMRP-USP), Brazil;; 8 Universidade de Campinas (UNICAMP), Brazil;; 9 Santa Casa de Belo Horizonte, Brazil;; 10 Hospital da Restauração de Recife (HR-Recife), Brazil;; 11 Hospital de Pediatría Dr. Juan P. Garrahan, Buenos Aires, Argentina;; 12 Centre d'Esclerosi Múltiple de Catalunya, Hospital Vall d'Hebron, Barcelona, Spain;; 13 Mellen Center for Multiple Sclerosis, Cleveland Clinic, OH;; 14 Hospital das Clínicas da Faculdade de Medicina da Universidade de São Paulo (HCFM-USP), Brazil; and; 15 Pontifícia Universidade Católica do Rio Grande do Sul (PUCRS), Porto Alegre, Brazil.

## Abstract

**Background and Objectives:**

Subspecialty exchange programs in neuroimmunology are scarce and have not been formally evaluated. We assessed participant-reported outcomes of the Neuroimmunology Exchange Scholarship Program (PROBEN), a 1-month immersive placement sponsored by the Brazilian Committee for Treatment and Research in Multiple Sclerosis, covering career trajectory, research engagement, and mentorship.

**Methods:**

Cross-sectional online survey of all 39 Brazilian neurology residents, fellows, and early-career neurologists who completed a PROBEN placement during the 3 most recent cycles (2023–2025) at 13 host centers (9 in Brazil, 4 abroad); 30 responded (77%). Outcomes were compared by career stage at placement (early career, residents or general neurologists, vs later stage, neuroimmunology fellows or neuroimmunologists) and by location (national vs international), using the Kruskal-Wallis, chi-square (Yates correction), and Fisher exact tests. Analyses were exploratory and unadjusted for multiple comparisons.

**Results:**

The mean age was 30 years (range 26–38); 18 respondents (60%) were early career and 18 (60%) trained at national centers. Satisfaction was high (17 of 30 [57%] excellent), all 30 would recommend the program, and 18 of 29 (62%, 95% CI 44–77) reported that it influenced their decision to continue in neuroimmunology, with no difference by location (*p* = 0.94). Perceived clinical (*p* = 0.71) and scientific (*p* = 0.46) knowledge gains were similar across settings. International placement was associated with more post-program conference presentations (9 of 12 vs 2 of 18; odds ratio 24.0, 95% CI 3.4–171.6; *p* = 0.001) and maintained mentor contact (11 of 12 vs 9 of 18; odds ratio 11.0, 95% CI 1.2–104.0; *p* = 0.02). Research activity (*p* = 0.009; epsilon-squared 0.23) and mentor engagement (*p* = 0.03; epsilon-squared 0.16) were higher among later-stage participants. Overall, 11 of 30 (37%) reported research output and 1 of 30 (3%) a completed or published manuscript.

**Discussion:**

Participants in this 1-month neuroimmunology exchange reported sustained subspecialty interest, ongoing mentorship, and academic engagement. Later career stage and international placement were associated with greater academic engagement in this cohort. Because participants were competitively selected and largely self-selected into placement location, these associations should not be read as causal effects of the program.

## Introduction

In an increasingly interconnected world, medical education exchange programs serve as a critical avenue for enhancing clinical expertise, research collaboration, and cross-cultural competency among health care professionals.^1,2^ These programs provide trainees with immersive learning experiences, exposing them to diverse patient populations, novel health care infrastructure, and cutting-edge medical technologies that may not be available in their home institutions.^2^ By fostering exchange of knowledge and professional networking, these initiatives contribute to building global capacity in specialized fields of medicine.^3,4^

In neurology, educational exchange programs have traditionally focused on early-career training, such as medical students and general neurology residents. These programs prioritize broad-based clinical skill development, offering exposure to diverse health care systems and neurologic conditions.^5^ While foundational neurology training through exchange programs has been widely studied, subspecialty-focused programs remain largely unexplored, particularly in neuroimmunology, a rapidly evolving field at the intersection of neurology and immunology.^6,7^

Neuroimmunology deals with highly complex, immune-mediated neurologic disorders such as multiple sclerosis (MS), neuromyelitis optica spectrum disorder (NMOSD), and autoimmune encephalitis.^6^ Specialized training in this field requires access to advanced imaging, biomarker research, immunotherapies, and multidisciplinary expertise, many of which are not readily available in low- and middle-income countries (LMICs).^8^ The limited access to cutting-edge diagnostic tools, disease-modifying therapies, and research infrastructure in these settings underscores the critical need for structured neuroimmunology exchange programs to bridge these gaps.^1,6^

Despite their potential benefits, subspecialization exchange programs in neuroimmunology face several challenges.^7^ Key barriers include disparities in training resources and research infrastructure, particularly between high-income countries and LMICs, limiting access to advanced diagnostics, immunotherapies, and cutting-edge research opportunities.^9^ In addition, trainees in certain regions may have limited exposure to rare and complex neuroimmunologic conditions, restricting hands-on clinical learning.^10^ Maintaining long-term mentorship and research collaborations postexchange also remains difficult, often because of geographic and institutional barriers.^2,11^ Ethical considerations, including issues of equity, power imbalances, and cultural adaptation, must also be carefully addressed to ensure that international training programs foster mutually beneficial and sustainable partnerships.^12^

These barriers are particularly relevant in Brazil, a continental country of more than 200 million inhabitants in which neuroimmunology expertise is unevenly distributed. Reported multiple sclerosis prevalence ranges from approximately 15 to 27 per 100,000 inhabitants across Brazilian regions, and a nationwide analysis of 2,974 patients in the BRANDO database documented substantial regional differences in health care access, with the longest intervals from symptom onset to diagnosis in the North and Northeast.^13^ The neurology workforce is similarly unequal: although the number of neurologists in Brazil grew substantially between 2010 and 2020, supply remains concentrated in the Southeast and South.^14^ Adult neurology residency lasts 3 years, the first of which is largely devoted to internal medicine rotations, and programs are themselves unevenly distributed across the country.^15^ Dedicated post-residency neuroimmunology training is concentrated in relatively few centers, so exposure to the subspecialty depends heavily on where a trainee happens to train. A national exchange scheme is therefore one of the few available mechanisms for extending subspecialty experience beyond a small number of established referral centers.

To address these gaps, the Brazilian Committee for Treatment and Research in Multiple Sclerosis (BCTRIMS) developed the Neuroimmunology Exchange Scholarship Program (PROBEN), a program designed to provide one-month immersive training experiences in leading national and international neuroimmunology centers. This study aims to evaluate the impact of PROBEN on participants' clinical training, research engagement, mentorship retention, and career trajectory. By analyzing the challenges and benefits of this program, we seek to provide actionable insights to optimize future neuroimmunology education initiatives, strengthen global partnerships, and enhance training accessibility worldwide.

## Methods

The program was established in 2016 as part of the BCTRIMS Youth League, an initiative by BCTRIMS to enhance education, foster hands-on clinical and research experience, and build a network of young neurologists and medical students interested in multiple sclerosis, neuromyelitis optica, and other neuroimmunology diseases.^16^ Applicants go through an annual selection process that includes current level of training, participation in national MS meetings, CV analysis, and letter of interest as the selection criteria. The program provides financial and administrative support, so the participants undertake a one-month exchange program in leading neuroimmunology institutions both in Brazil and abroad.

For readers unfamiliar with the Brazilian system, medical training begins directly after secondary school and comprises a 6-year undergraduate medical course, so that most physicians qualify at approximately 24 years of age. Adult neurology residency then lasts 3 years, the first of which is largely devoted to internal medicine rotations.^15^ Brazilian neurologists therefore typically complete residency at about 27 years of age, so the mean age of 30 years here corresponds to the final year of residency or the first years after it.

Applicants indicate preferred host institutions in their letter of interest, and the final placement is agreed between the applicant and the BCTRIMS scientific committee on the basis of the applicant's stated interests, host-center availability, and, for international placements, the applicant's ability to satisfy host-institution language, visa, and observership requirements. Placement location was therefore not randomly assigned and reflects participant preference and eligibility as well as program logistics, which is central to interpreting the comparisons between national and international placements reported below.

### Study Design and Participants

This study is a cross-sectional survey designed to assess the career impact of the PROBEN on its participants. Neurology residents, fellows, and trained neurologists who participated in the PROBEN program were invited to complete an online survey hosted on the Research Electronic Data Capture (REDCap) platform. Participants were recruited via email and professional networks affiliated with BCTRIMS. Between 2016 and 2025, 126 physicians were selected for PROBEN. To limit recall bias and to ensure that respondents could reliably report on program content and immediate post-program outcomes, the survey was restricted to the 39 individuals who completed the program during the 3 most recent cycles (2023–2025). All 39 eligible participants were invited; 30 completed the survey (response rate 77%). REDCap was configured to accept one response per invited participant, and responses were anonymized at export.

### Survey Instrument

The survey consisted of structured multiple-choice and open-ended questions designed to capture various aspects of participants' professional trajectories, program characteristics, and perceived outcomes. The questionnaire was divided into the following sections: The questionnaire was developed de novo by the study team because no validated instrument for evaluating subspecialty neurology exchange programs was identified. Items were drafted from the published literature on global health electives and on neuroimmunology fellowship training, reviewed for content and clarity by BCTRIMS Youth League faculty, and pilot tested with former PROBEN participants who were not part of the study sample, with wording revised accordingly. The instrument was not subjected to formal psychometric validation, no composite scale scores were derived, and each item was analyzed individually. The full questionnaire is provided as eAppendix 1. Reporting follows the Consensus-Based Checklist for Reporting of Survey Studies (CROSS).^17^

#### Demographics and Career Achievements

In this section, questions included the year in which the PROBEN program was completed; age at the time of the program; professional role during the program (e.g., junior neurology resident, senior resident, neuroimmunology fellow, general neurologist, neurologist specializing in MS/neuroimmunology); highest academic degree obtained (Master's, Doctorate, Postdoctoral training); current workplace and primary clinical or research focus.

#### Program Characteristics

The survey enquired about the location of the internship (national or international); specific institution where the internship took place; duration of the internship (less than 30 days, exactly 30 days, or more than 30 days); nature of activities performed (e.g., outpatient care, inpatient care, clinical observation, hands-on experience, clinical or basic research, case discussions); level of autonomy granted during the internship (observational vs patient involvement); and perceived receptivity and hospitality from the host institution and mentors.

#### Perceived Gains From the Experience

The survey also contained questions on the acquisition of new knowledge (e.g., emerging research, novel diagnostic and treatment strategies); development of new technical skills (e.g., neurologic examination techniques, procedures); growth in scientific understanding and career insights; exposure to new work environments and different healthcare systems; strengthened networking with professionals in neuroimmunology; and new opportunities for national and international research collaborations.

#### Research and Academic Outcomes

This included whether the internship led to research outputs such as conference attendance (e.g., BCTRIMS Annual Meeting, national or international conferences); abstract submissions; poster presentations; manuscripts in progress or published articles; and level of involvement in research activities during the internship.

#### Long-Term Impact on Career

Long-term impact was assessed based on whether the experience influenced career choices; the proportion of neuroimmunology patients seen in current practice; whether the internship influenced the decision to pursue a career in neuroimmunology; continued professional relationships with mentors postinternship; and overall impact on professional and personal growth (e.g., cultural exchange, work-life balance adjustments, expanded professional opportunities).

### Statistical Analysis

Survey responses were exported from REDCap and analyzed in R (R Core Team).^18^ Continuous variables are summarized as means with ranges and categorical variables as counts and percentages. Because a small number of items were left blank, the denominator is stated alongside each proportion and percentages are calculated among respondents who answered that item. Two groupings were compared: career stage at the time of the exchange (early-career participants, that is, neurology residents or general neurologists, vs later-stage participants, that is, neuroimmunology fellows or neuroimmunologists) and placement location (national vs international). Associations between these groupings and categorical outcomes were assessed with the Pearson χ^2^ test applying the Yates continuity correction and with the Fisher exact test when expected cell counts were fewer than 5. Ordinal outcomes, including level of research activity and perceived impact, were compared using the Kruskal-Wallis test. To convey the magnitude and the precision of associations rather than statistical significance alone, we report odds ratios with 95% CIs for 2 × 2 comparisons, the phi coefficient for chi-square comparisons, and epsilon-squared for Kruskal-Wallis comparisons; 95% CIs for single proportions were calculated by the Wilson method. All tests were 2-sided, and a *p* value below 0.05 was considered statistically significant. The analyses were exploratory and hypothesis-generating: multiple unadjusted comparisons were performed in a sample of 30 respondents, no correction for multiplicity was applied, and the resulting inflation of type I error means that individual *p* values near the 0.05 threshold should be interpreted with caution. Because the study was single-arm and observational, all comparisons describe associations within this cohort and not effects attributable to the program.

### Standard Protocol Approvals, Registrations, and Patient Consents

This study was approved by the local institutional review board of PUC-RS (Porto Alegre, Brazil)–approval number 6848279. Because this study involved a voluntary, anonymized survey of program participants and did not involve patients or patient data, written informed consent was not required; completion of the survey implied consent to participate, and all respondents were informed of the study's purpose and the voluntary nature of participation. No patient-identifiable information was collected.

### Data Availability

The deidentified individual participant data underlying the results reported in this article, together with the data dictionary and the survey instrument, will be made available to qualified investigators whose proposed use has been approved by an independent review committee. Proposals should be directed to the corresponding author and will require a signed data access agreement. Data will be available beginning 12 months and ending 5 years after article publication. The study protocol and statistical analysis plan will be shared on the same terms.

## Results

From 2016 to 2025, PROBEN received 333 applications. In total, 126 physicians were selected (approval rate of 38%). A total of 13 centers of expertise participated in the program: 9 in Brazil and 4 international. The present analysis is based on the 39 participants from the 2023–2025 cycles, of whom 30 responded (77%).

The mean age of respondents was 30 years (range 26–38). Fifteen respondents (50%) were neurology residents (13 senior and 2 junior), 11 (37%) were early-career neuroimmunologists, 3 (10%) were general neurologists, and 1 (3%) was a neuroimmunology fellow; by the prespecified grouping, 18 (60%) were early career and 12 (40%) later stage at the time of the placement. Prior academic attainment and engagement were substantial among those answering these items: 11 of 21 (52%) had completed a master's degree in neurology and 4 of 21 (19%) a doctorate, 14 of 21 (67%) participated in national or local research groups, and 5 of 21 (24%) reported international research collaborations. Leadership experience was also common, with 11 of 19 (58%) having organized local events, 10 of 19 (53%) national events, and 14 of 19 (74%) BCTRIMS events.

The placement lasted the planned 30 days for 25 participants (83%), fewer than 30 days for 4 (13%), and more than 30 days for 1 (3%). Eighteen participants (60%) trained at national institutions and 12 (40%) at international centers. Clinical exposure was extensive: all 30 (100%) attended outpatient clinics and 15 (50%) also observed inpatient diagnosis and management. Case discussions and lectures were attended by 27 participants (90%), and 3 (10%) took part in activities focused on clinical or basic science research.

Autonomy varied: 20 of 29 respondents (69%) described an observership role and 9 of 29 (31%) reported some degree of supervised patient care, the latter exclusively at national centers, reflecting licensing and visa restrictions at international sites. The financial cost of participation varied substantially, with 12 of 30 (40%) spending more than 10,000 Brazilian reais (approximately US $1,700 at the time of the placement) and 11 of 30 (37%) spending between 1,000 and 5,000 reais (approximately US $170–860).

Participants reported predominantly positive experiences: 25 of 30 (83%) cited professional growth, 18 of 30 (60%) personal development, and 15 of 30 (50%) acquisition of new clinical knowledge. Receptivity at the host institution was rated excellent by 24 of 30 (80%) and the mentorship experience by 17 of 29 (59%), and 17 of 30 (57%) reported strong scientific and technical skill advancement.

Nine of 29 respondents for that question (31%) reported slight language difficulties. Overall satisfaction was high, with 17 of 30 (57%) rating the experience as excellent and 11 of 30 (37%) as very good. All 30 participants (100%, 95% CI 89–100) would recommend the program and 27 of 30 (90%) would choose to participate again. Eighteen of 29 respondents (62%, 95% CI 44–77) reported that the placement influenced their decision to continue working in neuroimmunology.

Eleven of 30 respondents (37%, 95% CI 22–55) reported research output arising from the placement, including presentations at BCTRIMS ECHO and at the BCTRIMS Annual Meeting, whereas 1 of 30 (3%, 95% CI 1–17) reported a completed or published manuscript. The placement also fostered networking: 26 of 30 (87%) made professional connections, 21 of 30 (70%) formed new friendships, and 23 of 30 (77%) described cultural growth. Fifteen of 30 (50%) reported a moderate impact on their career and 13 of 30 (43%) a high professional impact.

### Career Stage

Among the 13 senior residents, 11 (85%) undertook national placements and 2 (15%) international placements. Three had obtained a master's degree in neurology and 2 a doctorate. Five (38%) were involved in local or national research groups and 4 (31%) in international research collaborations. No senior resident reported presenting at an international conference, 1 presented at a national congress, and none reported an ongoing or completed publication; 12 (92%) stated that they did not generate a research project or presentation during the placement. Regarding mentorship, 1 senior resident maintained frequent contact with the host mentor, 5 had occasional contact, and 7 reported no ongoing communication.

Research activity differed by career stage, with later-stage participants reporting higher levels of research engagement than early-career participants (Kruskal-Wallis test, *p* = 0.009; epsilon-squared 0.23). Mentor engagement was likewise higher among later-stage participants (Kruskal-Wallis test, *p* = 0.033; epsilon-squared 0.16). A χ^2^ test with the Yates continuity correction also showed differences across personal growth, professional development, and scientific output (*p* = 0.005; Figure 1). The direction was not uniform: scientific output was reported by 2 of 18 early-career vs 9 of 12 later-stage participants, whereas personal growth was reported by 15 of 18 vs 3 of 12. Because career stage and placement location were closely related in this cohort, with 11 of 13 senior residents placed nationally, these 2 comparisons cannot be disentangled from one another in a sample of this size.

**Figure 1:**
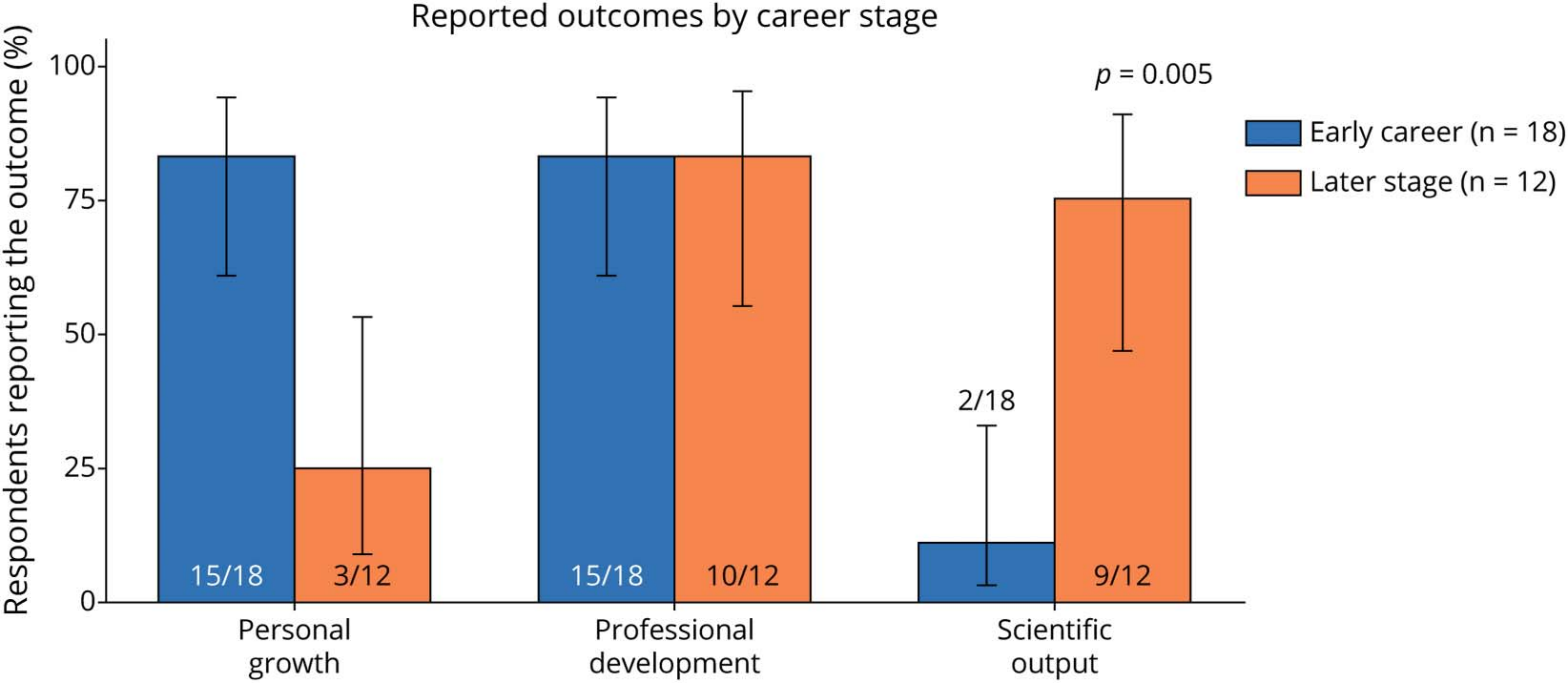
Reported Outcomes by Career Stage Graph shows the distribution of responses to 3 items, personal growth, professional development, and scientific output, among early-career participants (neurology residents or general neurologists, n = 18) and later-stage participants (neuroimmunology fellows or neuroimmunologists, n = 12). Each item was recorded as reported or not reported, so the density in each violin concentrates toward the 2 ends of the scale rather than forming a continuous curve. Individual responses are overlaid as jittered points; the white diamond marks the proportion reporting the outcome and the vertical line its 95% Wilson confidence interval; counts are printed above each violin. Comparison across the 3 outcomes by χ^2^ test with the Yates continuity correction, *p* = 0.005.

### National and International Programs

Participants in national placements (n = 18) had broad exposure to outpatient neurology, with all 18 (100%) attending outpatient follow-up clinics. Inpatient exposure was more limited, reported by 5 (28%). Research involvement was low, with 1 participant (6%) engaged in clinical research and 1 (6%) in basic research. Case discussions and lectures were attended by 17 (94%).

Regarding academic output in the national group, 1 participant (6%) presented at a national conference and none reported an international conference presentation or a publication. One participant took part in the BCTRIMS ECHO project as a result of the placement. Sixteen (89%) stated that they did not generate a research project or presentation from the experience, in some cases because host programs limited the ability of an observer to undertake research for regulatory reasons. Mentorship contact varied: 1 participant (6%) maintained frequent contact, 8 (44%) occasional contact, and 9 (50%) were no longer in touch.

Participants in international placements (n = 12) reported a more balanced experience across outpatient and inpatient settings, with all 12 (100%) attending outpatient clinics and 10 (83%) involved in inpatient care. Research participation was higher, with 2 (17%) involved in clinical research and 2 (17%) in basic research. Attendance at case discussions and lectures was 10 of 12 (83%), compared with 17 of 18 (94%) at national centers.

Academic engagement was greater in the international group, in which 6 participants (50%) presented at the BCTRIMS ECHO project, 5 (42%) at the BCTRIMS Annual Meeting, and 3 (25%) at other national conferences. One participant reported a research project in progress, and none had published an article arising from a collaborative research project. Mentorship engagement was also stronger: 2 participants (17%) maintained frequent mentor contact, 9 (75%) occasional contact, and 1 (8%) had lost contact.

Placement location was associated with post-program conference presentations (9 of 12 international vs 2 of 18 national; odds ratio 24.0, 95% CI 3.4–171.6; Fisher exact test, *p* = 0.001) and with maintained contact with the host mentor (11 of 12 vs 9 of 18; odds ratio 11.0, 95% CI 1.2–104.0; Fisher exact test, *p* = 0.02). The width of these confidence intervals reflects the small number of participants in each group and indicates that the magnitude of the association is estimated imprecisely. Chi-square tests with the Yates continuity correction also showed differences by placement location in personal growth, professional growth, and scientific output (*p* = 0.002; Figure 2). No association was detected between placement location and self-reported clinical knowledge gained (*p* = 0.71; phi = 0.07) or scientific knowledge gained (*p* = 0.46; phi = 0.13).

**Figure 2:**
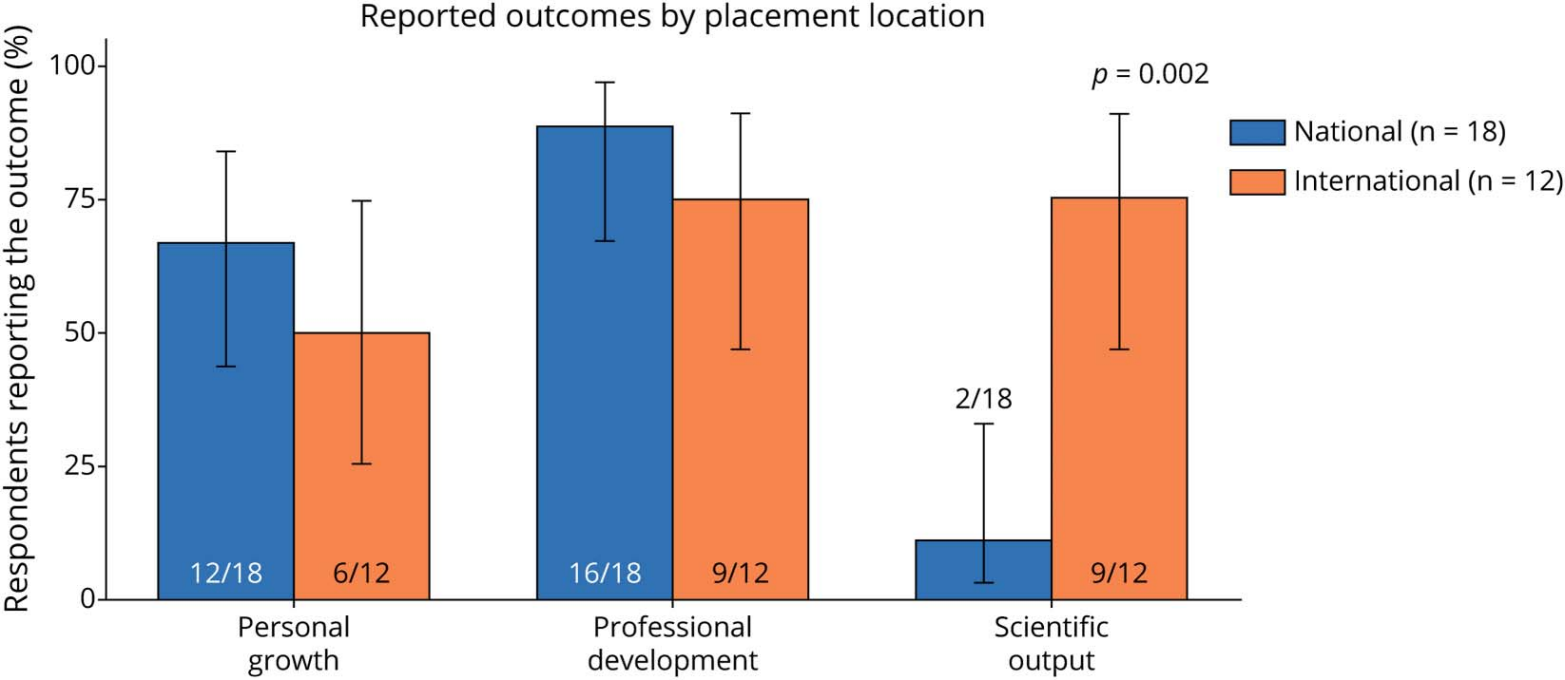
Reported Outcomes by Placement Location Graph shows the distribution of responses to the same 3 items among participants at national (n = 18) and international (n = 12) host centers, plotted as in Figure 1. Comparison by χ^2^ test with the Yates continuity correction, *p* = 0.002.

Kruskal-Wallis tests showed no detectable difference by placement location in the perceived impact on pursuing neuroimmunology (*p* = 0.94; epsilon-squared less than 0.01) or on professional life (*p* = 0.39; epsilon-squared 0.03). A difference in perceived personal life impact did not reach the significance threshold (*p* = 0.07; epsilon-squared 0.12). The Fisher exact test showed no association between placement location and research involvement during the placement (*p* = 0.39). Given the number of unadjusted comparisons performed in 30 respondents, these findings indicate an absence of a detectable difference rather than evidence of equivalence.

## Discussion

Our study describes participant-reported outcomes of a national neuroimmunology exchange program across research engagement, mentorship, professional development, and career trajectory and examines how these differ by career stage and training setting. Understanding the challenges of subspecialty exchange programs, particularly in fields like neuroimmunology, is crucial for several reasons. Neuroimmunology is a highly specialized area within neurology that requires specific expertise, resources, and training environments, which may not always be available in every institution.^19^ Without training in an expert center, MS/neuroimmunology instruction risks offering trainees experiences that are less comprehensive or impactful, potentially limiting their ability to gain the depth of knowledge required for diagnosis and management in these areas.^7,20^ Effective exchange programs must ensure that trainees are placed in settings where they can engage with cases that allow for practical learning and the development of diagnostic and therapeutic skills that are critical in neuroimmunology.^6,10^

A principal finding is that 18 of 29 respondents (62%, 95% CI 44–77) reported that the placement influenced their decision to continue in neuroimmunology, with no difference detected between participants at national and international centers.

International placement was associated with more post-program conference presentations and with a higher likelihood of maintained mentor contact. These associations should not be read as effects of the international setting itself. Participants who undertook international placements were, as a group, at a later career stage, were more likely to be already engaged in research, and had self-selected into placements that required language proficiency and the ability to meet host-institution visa and observership requirements. Greater protected academic time, a preexisting research orientation, and language ability are therefore plausible alternative explanations for the difference we observed, and the present design cannot distinguish them from any contribution of the host setting. A sample of 30 does not support multivariable adjustment to separate these influences. Neuroimmunology as a subspecialty requires not only clinical experience but also a deep understanding of the latest research and advancements in immunotherapies and biomarkers.^6^ Exchange programs focused on neuroimmunology must provide access to cutting-edge research and allow for collaboration with specialists in the field.^6^ The challenges here lie in ensuring that both the sending and receiving institutions are well-equipped to offer training in this rapidly advancing field, which can be difficult in settings that may have limited academic time.^6,8^

Post-program research activity was greater among participants who had already completed residency, and a similar pattern was seen for the maintenance of long-term mentor-mentee relationships. Within this cohort, therefore, later-stage participants appeared to have greater academic engagement. We read this as a signal that placements intended to generate scholarly output may be most productive when offered to participants who already have protected time and a defined research question, rather than as evidence that later-stage placement maximizes academic return. Residents may derive different and equally legitimate benefits, including exposure to subspecialty practice, calibration of clinical reasoning, and clarification of career direction, that the outcome measures used here were not designed to capture.

Language warrants specific comment. Nearly a third of respondents reported at least slight language difficulty, which is a real obstacle to full participation in clinical reasoning, case discussion, and research at an international host center, and which compounds the observership restrictions already imposed by licensing and visa rules. At the same time, the language demand is part of what the program transmits. Participants who become able to work across Portuguese, Spanish, and English are subsequently positioned to bring international guidelines, trial literature, and collaborative networks back into Brazilian practice and to teach them locally. Programs might therefore offer targeted language preparation before international placements and pair participants with bilingual host mentors, rather than treating language proficiency solely as a selection criterion.

Table summarizes the principal challenges encountered across the PROBEN program, the lessons drawn from them, and the responses we would recommend, in 8 domains from resource disparities and case mix through mentorship and reintegration to the assessment of educational outcomes. These observations reflect the collective experience of participants, mentors, and coordinators together with free-text survey responses; the recommendations are consensus-based, were not formally tested, and should not be read as empirical findings of this study.

**Table:** Challenges Encountered in the PROBEN Neuroimmunology Exchange Program, With Lessons Learned and Corresponding Recommendations

Challenge domain	Observed in PROBEN	Key recommendations	Suggested indicator of success
Resource and infrastructure disparities	Marked asymmetry in diagnostic capacity: 3T MRI with standardized MS protocols, CSF isoelectric focusing, AQP4-IgG and MOG-IgG cell-based assays, and neurofilament light were routine at some hosts and unavailable at home. Reverse placements taught diagnosis from a compressed panel	Frame resource constraint as a teachable skill, training which test, for which patient, at what cost. Have trainees draft a minimum viable diagnostic pathway for the home center before departure and revise it on return	Diagnostic protocols, MRI sequences, or assays adopted at the home center within 12 mo
Epidemiology and case mix	Latin American sites saw proportionally more NMOSD and MOGAD and routinely excluded HTLV-1-associated myelopathy, neuroschistosomiasis, and tuberculous or syphilitic myelitis. Sites abroad offered progressive and pediatric-onset MS and trial populations	Select sites for complementary case mix rather than prestige. Require a logged set of cases unavailable at home, and build reciprocal teaching so visiting trainees present home-region material	Case log completeness; at least one trainee-led case conference delivered at the host site
Therapy access and treatment paradigms	Treatment algorithms diverged more than diagnostic ones: escalation vs early high-efficacy strategies, rituximab vs ocrelizumab, aHSCT access, and thresholds for treating RIS varied by site and payer. Trainees struggled to reconcile guideline-concordant care with local reimbursement	Teach the evidence and the constraint separately, distinguishing what the trial showed from what the health system funds. Include a session comparing host and home reimbursement and regulatory pathways	A locally adapted treatment algorithm or formulary submission; participation in home protocol revision
Mentorship and supervision	Mentorship quality tracked with trainee-reported value and sustained engagement more closely than site resources. Absent or overcommitted mentors led to absorption into service work; explicit, protected mentorship often produced relationships that outlasted the placement	Name one accountable host mentor before confirming the placement, with defined contact frequency. Keep the home mentor active throughout, and schedule structured midpoint and exit feedback	Proportion of trainee-mentor dyads still in contact or collaborating at 12 and 24 mo
Administrative, regulatory, and financial barriers	The commonest reason a planned placement failed was administrative rather than academic: visa timelines, licensing or observership status, occupational health requirements, and cost. Funding constraints fell disproportionately on trainees from lower-resource centers	Publish a checklist with realistic lead times and begin 6–12 mo in advance. Secure need-based travel funding with transparent criteria, and designate an administrative coordinator rather than leaving logistics to trainees	Proportion of accepted trainees completing the placement; time from acceptance to departure; proportion of need-based awards
Reintegration and knowledge translation	Learning frequently failed at the point of return. Trainees came home with concrete proposals and met institutional inertia, absent funding, or no forum in which to present, so individual learning did not become institutional capability	Make a defined re-entry deliverable a condition of the award. Engage home leadership before departure, convene an annual meeting at which returning trainees present, and track implementation at 6 and 12 mo	Proportion completing a re-entry deliverable; changes implemented at the home institution at 12 mo
Sustainability, reciprocity, and equity	Exchange flow was asymmetric, running from lower- to higher-resource centers	Build bidirectionality into the program design and monitor the flow explicitly. Prioritize projects that address a problem defined by the lower-resource partner	Ratio of inbound to outbound placements; joint publications with a home-center first or senior author; trainees practicing in their home region at 24 mo
Assessment of educational outcomes	Evaluation relied largely on end-of-placement satisfaction, which was uniformly high and therefore discriminated poorly between placements of clearly different educational value	Move to competency-based assessment using entrustable professional activities, case-based discussion, and workplace-based observation. Prespecify the outcomes the program intends to move, and report null findings and placements that did not work	Proportion of placements with a completed competency-based assessment; completeness of 12-mo follow-up

Abbreviations: aHSCT = autologous hematopoietic stem cell transplantation; AQP4-IgG = aquaporin-4 immunoglobulin G; HTLV-1 = human T-lymphotropic virus type 1; MOGAD = myelin oligodendrocyte glycoprotein antibody–associated disease; MRI = magnetic resonance imaging; MS = multiple sclerosis; NMOSD = neuromyelitis optica spectrum disorder; PROBEN = Programa Brasileiro de Estagio em Neuroimunologia; RIS = radiologically isolated syndrome.

Observations reflect the collective experience of program participants, mentors, and coordinators, together with free-text survey responses; recommendations are consensus-based and were not formally tested.

Our study has important limitations. First, the design is cross-sectional, single-arm, and observational. There was no comparison group and no measurement before the placement, so all findings describe associations and participant perceptions rather than outcomes caused by the program. Second, selection bias is likely to be substantial. PROBEN admits approximately 38% of applicants on the basis of training level, prior meeting participation, curriculum vitae, and letter of interest, so participants are by design unusually motivated; the research output and mentorship retention we observed may reflect the characteristics of those selected at least as much as the experience they received. Third, response bias may act in the same direction: 9 of 39 eligible participants did not respond, and those with less favorable experiences or with less continued involvement in neuroimmunology may have been less likely to reply, which would inflate the positive ratings. Fourth, outcomes were self-reported and retrospective, without independent verification of publications or presentations, introducing recall and social desirability bias. Fifth, placement location was not randomly assigned; participants expressed preferences and international placements required language and visa eligibility, so comparisons between national and international groups are confounded by career stage, prior research engagement, and language proficiency, and the 2 groupings could not be separated statistically in a sample of 30. Sixth, the analyses were exploratory and multiple unadjusted comparisons were performed, so some findings may be false positives, and the wide confidence intervals around our effect estimates indicate their imprecision. Seventh, host programs varied in structure, autonomy, and research opportunity, which we did not characterize in a standardized way. Finally, because the survey was restricted to the 3 most recent cycles, the findings may not generalize to earlier cohorts and follow-up was short; the low proportion reporting a completed manuscript likely underestimates eventual scholarly output, since projects begun during the exchange may remain in progress. Conversely, restricting the sample to recent participants reduced recall bias regarding program content and mentorship.

Several questions follow directly from these limitations. Prospective designs with measurement before and after the placement, a comparison group of unsuccessful applicants or of contemporaneous nonparticipants, and longer follow-up would allow the contribution of the program to be distinguished from the trajectory of highly motivated trainees. Outcome measurement should also extend beyond the individual participant to the propagation of skills: whether former participants subsequently teach, supervise, or mentor others; whether they establish or strengthen neuroimmunology services in underserved regions; and whether host relationships generate collaborations that persist beyond the placement would be more informative indicators of program value than counts of presentations. Objective verification of scholarly output and analysis stratified by career stage would further strengthen future evaluations.

In summary, participants in a structured 1-month neuroimmunology exchange reported sustained subspecialty interest, ongoing mentorship, and academic engagement, with greater academic engagement among those at a later career stage and those placed internationally. These observations are hypothesis-generating rather than causal, but they suggest that matching participants to program goals, emphasizing diagnostic and management skills for earlier-stage trainees and research and academic development for those with protected time, may help exchange programs make the best use of a scarce and costly resource. Attention to resource disparities, preparation, mentorship, and sustainability will be important if neuroimmunology education is to expand equitably worldwide.

## Glossary

BCTRIMS: Brazilian Committee for Treatment and Research in Multiple Sclerosis
LMICs: low- and middle-income countries
MS: multiple sclerosis
NMOSD: neuromyelitis optica spectrum disorder
PROBEN: Neuroimmunology Exchange Scholarship Program
REDCap: Research Electronic Data Capture
